# A multilevel data integration resource for breast cancer study

**DOI:** 10.1186/1752-0509-4-76

**Published:** 2010-06-03

**Authors:** Ettore Mosca, Roberta Alfieri, Ivan Merelli, Federica Viti, Andrea Calabria, Luciano Milanesi

**Affiliations:** 1 Institute for Biomedical Technologies, National Research Council, Segrate (Milan), Italy

## Abstract

**Background:**

Breast cancer is one of the most common cancer types. Due to the complexity of this disease, it is important to face its study with an integrated and multilevel approach, from genes, transcripts and proteins to molecular networks, cell populations and tissues. According to the systems biology perspective, the biological functions arise from complex networks: in this context, concepts like molecular pathways, protein-protein interactions (PPIs), mathematical models and ontologies play an important role for dissecting such complexity.

**Results:**

In this work we present the Genes-to-Systems Breast Cancer (G2SBC) Database, a resource which integrates data about genes, transcripts and proteins reported in literature as altered in breast cancer cells. Beside the data integration, we provide an ontology based query system and analysis tools related to intracellular pathways, PPIs, protein structure and systems modelling, in order to facilitate the study of breast cancer using a multilevel perspective. The resource is available at the URL http://www.itb.cnr.it/breastcancer.

**Conclusions:**

The G2SBC Database represents a systems biology oriented data integration approach devoted to breast cancer. By means of the analysis capabilities provided by the web interface, it is possible to overcome the limits of reductionist resources, enabling predictions that can lead to new experiments.

## Background

Cancer is a complex disease in which both genomic and environmental factors affect the functioning of the molecular circuits leading to the so-called acquired capabilities of cancer [1]. Due to its complexity, it is important to face the study of cancer exploiting an integrated and multilevel approach, ranging from genes, transcripts and proteins found altered in cancer cells, to whole biological systems, represented by molecular pathways and cell populations. The study of complex systems in biology is addressed by systems biology, which is providing new opportunities in cancer research [2]. Suitable examples are the study of regulatory and signal transduction networks, mostly affected by genomic mutations leading to cancer, and the analysis of cell populations dynamics.

To realise a multilevel and systems oriented approach about a disease, it is crucial to collect and integrate data stored in several dedicated resources. Currently, this process is characterised by some issues. First, data required to realise this perspective are still sparse on the web: despite some existing databases (such as those developed by the NCBI and the EBI) collect data from several projects, data provided by specific resources dedicated to particular pathologies are not yet integrated and therefore are difficult to exploit. Moreover, the accessible information is by far too heterogeneous: for example, some resources make their content available relying on identifiers that do not match directly. Another issue concerns the relevance of data produced by using high-throughput technologies, which represent a useful source of information and, therefore, are essential in a data integration approach: this is the case, for instance, of protein-protein interactions (PPIs) data, that enable the study of cellular networks structure by means of graph theory approaches. Lastly, even if several mathematical models have been developed in the cancer research field, many of them are not coded in standard languages and thus they are not directly available for simulations. In this systems biology perspective, we chose to focus our research on one of the most common cancer types, the breast cancer, which has a high impact on the population and is studied within our institute (see, for instance, [3-5]).

Generic as well as scientifically relevant resources exist concerning this pathology. "Oncomine" [6] was developed for cancer gene expression analysis; "The Tumour Gene Family of Databases" [7] contains information about genes which are targets for cancer-causing mutations; the "BreastCancerDatabase" [8] collects molecular alterations associated with breast cancer; the "Breast Cancer Information Core Database" [9] stores mutations of main breast cancer genes. However, the scientific community lacks easily accessible data dealing with breast cancer in a multilevel context, including molecules, molecular networks, cells and tissues.

To fill this gap we developed the Genes-to-Systems Breast Cancer (G2SBC) Database. This resource realises the integration of information concerning molecular components related to breast cancer and the overlying molecular and cellular layers, even providing a series of tools for the analysis of the available data.

## Construction and Content

**Figure 1:**
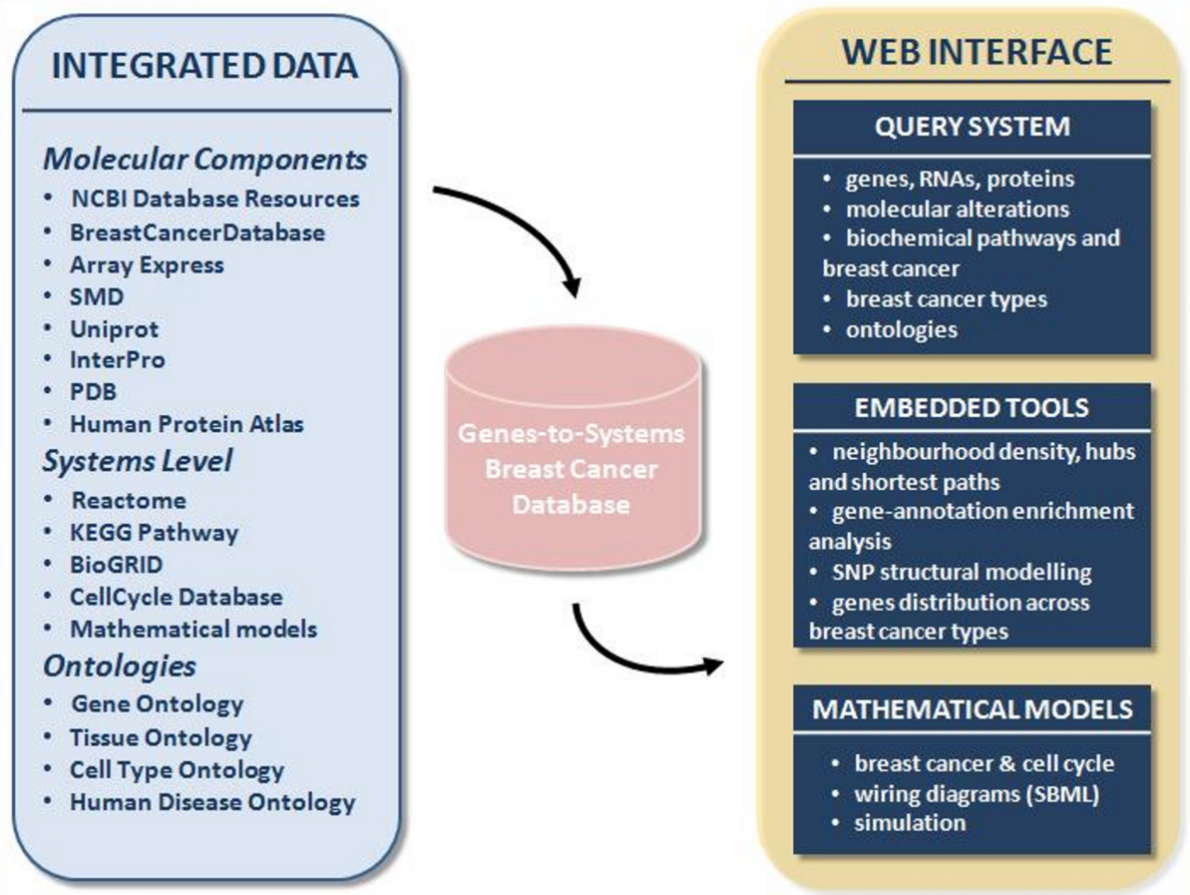
**Architecture of the G2SBC Database**. The G2SBC Database is the result of a multilevel data integration approach. The web interface provides a query system, a series of tools for the analysis of the contents and a mathematical models section.

The G2SBC Database relies on a MySQL server. The database structure follows a data warehouse approach, which consists in collecting and formatting heterogeneous data from different sources, in order to make them accessible by the scientific community through a unified query schema using a web interface. This approach is typical of data integration, while normalised databases, designed to support data integrity, are widely used to maintain primary resources. From the data integration point of view, a series of perl scripts have been developed to retrieve different datasets from remote and sparse data sources. The architecture of the G2SBC Database is illustrated in Figure 1. The G2SBC Database maintains data about genes, transcripts, proteins, molecular and cellular systems, and mathematical models related to the breast cancer pathology. The associations between genes and breast cancer is supported by a number of molecular evidences derived from literature: we refer to genes having at least one evidence as "breast cancer genes" and to proteins as "breast cancer proteins". Currently, the G2SBC Database provides literature based evidences of molecular alterations for more than 2000 human genes. Each molecular alteration is reported along with a reference to the paper in which the experimental identification is described. The complete list of molecular alterations considered at genome, transcriptome and proteome levels is reported in Table 1. Note that the G2SBC Database contains data about all the human genes, since such knowledge is required to perform the analyses provided by the tools available through the web interface, e.g. the tools based on the PPIs.

**Table 1:** Number of genes associated with the listed types of molecular alteration

*Genome*		Cell line	Clinical
	Amplification	25	24
	Deletion	5	16
	Insertion	2	8
	Methylation/Acetylation	18	23
	Single Nucleotide Polymorphism	3	21
	Transition	14	39
	Translocation	3	1
	Transversion	16	47
*Transcriptome*			

	Expression Level	88	2027
	Splice Variant	28	17
*Proteome*			

	Activity	8	12
	Altered Interaction	16	3
	Altered Isoform Splice Variant	10	9
	Altered Localisation	9	31
	Amino Acid Change	6	31
	Deletion	0	2
	Expression Level	36	117
	Post Translational Modification	13	4

*Genes affected by at least one molecular alteration*		*2238*	

Data has been retrieved from literature, both relying on a manual curation process and exploiting automated methods for data integration from datasets available on the web [8,10-22].

### Molecular components data

Breast cancer genes are annotated by gene symbol, description, aliases and sequences. The list of molecular alterations found in breast cancer spans the genome, transcriptome and proteome layers and comes from both clinical data and cell line experiments. Gene characterisation is enriched with information about the related Single Nucleotide Polymorphisms (SNPs), downloaded from dbSNP [10]. SNPs are ordered according to their distribution along the gene and polymorphisms occurring within exons are highlighted in order to allow the structural modelling of the resulting protein.

Gene products have been collected as list of mRNAs sequences and related protein isoforms according to the NCBI RefSeq annotations (NCBI Nucleotide [10]). Gene expression information is supported by a link towards the Gene Expression Atlas [14] report, where the over/under expression of the current gene in a set of conditions (including different diseases) is listed. Moreover, information about expression profiles similarity has been collected from a study of co-expression analysis [5], which focused on a dataset of breast primary tumours derived from the Stanford Microarray Database [15]. Each gene is also associated with a list of microRNAs (microRNA.org [22]) which target the gene transcripts.

Data about proteins include all the identifiers suitable to download the related sequences, functional domains according to InterPro [12], structural models from the Protein Data Bank (PDB) [13] and drugs available to affect their function [8]. Moreover, tissue images, showing the expression and the localisation of proteins in a large variety of human breast tissues, have been collected from the Human Protein Atlas (HPA) [21]. This section maintains images obtained in different conditions of breast organ (normal breast, ductal breast cancer, lobular *in situ *breast cancer, lobular breast cancer, malignant neoplasy, hyperplasia). Each image is associated with information concerning the protein detected on the tissue, its spatial localisation (nuclei or cytoplasm/membrane), some patients clinical information, staining intensity (negative, weak, moderate or strong) and quantity of stained cells (rare, < 25%, 25-75% or >75%). Similarly to what has been done in the HPA database, values of the cartesian product of intensity and quantity (i.e. (negative, rare), (negative, < 25%), (negative, 25-75%), ..., (strong, > 75%)) have been mapped to natural numbers belonging to the set {1, 2, ..., 16}. These numbers represent a score for the protein expression, where 1 is associated with (negative, rare) and 16 with (strong, > 75%). Following this operation it has been possible to calculate average protein expression and to infer protein differential expression between breast cancer tissues and normal tissues as difference between two protein expression scores.

### Systems level data

Considering the molecular systems layer, the G2SBC Database lists the biochemical pathways (KEGG [16], Reactome [17] and Gene Ontology (GO) Biological Processes (BP) [20]) - with the term "biochemical pathway" we refer to any type of molecular circuit, i.e. metabolic, signalling and gene regulatory, since all of them are based on biochemical processes such as biochemical reactions, proteins association and dissociation - and PPIs (collected from BioGRID [18]) that complement information about biochemical pathways.

PPIs data have been used to create a PPIs network that has been analysed using concepts derived from the graph theory. In particular, clustering coefficients (a measure of the neighbourhood density), all-pairs shortest paths (a shortest path in a graph is the path connecting two vertices such that the sum of the weights of its edges is minimised) and their lengths (the number of edges that belong to the path) have been calculated. Other interesting information related to molecular systems is represented by the association of the breast cancer genes with the cell cycle process (CellCycle Database [19,23]), including the set of mathematical models available for this process.

Considering the cellular systems level, the database includes data about breast cancer types, tissue images and mathematical models (mostly based on ordinary differential equations) available in literature, which focus on carcinogenesis, tumour growth and tumour response.

### Ontologies

To provide a standard framework for data integration and to enhance a systemic view of breast cancer information, a rich ontology layer underlies the database structure. Where available, genes are annotated using the GO and biochemical pathways by the KEGG Pathway [16] ontology (derived from the hierarchical organisation of KEGG pathways). This approach not only allows the availability of a commonly recognised vocabulary, that promotes data sharing and information querying, but also increases the performance of statistical and analytical studies. Indeed, the graphs *G*_*i *_= (*N*_*i*_,*E*_*i*_) (where *N*_*i *_is the set of vocabulary terms, *E*_*i *_is the set of edges between terms and *i *identifies a particular ontology), that undergo the hierarchically structured terms, represent a crucial instrument to shed light on relationships between biological components, thus performing more accurate queries and even promoting the deduction of new relations. Ontologies have been exploited even to better define the scientific context of the developed resource. Human disease ontology [24], BRENDA tissue ontology [25] and cell type ontology [26] are included in the ontology section: these ontologies can be browsed by means of the Ontology Lookup Service [27], an EBI tool that provides a web service interface to query multiple ontologies, and allow the localisation of a particular element (such as breast cancer, mammary gland and mammary gland cells) among the hierarchically organised elements of the same ontology (human diseases, human tissues and human cell types respectively).

## Utility and Discussion

The web interface of the G2SBC Database (implemented employing PHP and JavaScript languages), provides a series of tools to show and analyse data collected in the resource. An extensive help section is available through the left side menu of the web site and shows detailed examples on how to explore the database content and how to exploit the tools integrated in the resource: use cases are described for illustrating what kind of predictions are allowed by the G2SBC database.

The web site pages are grouped into three main sections, listed in the left side menu. The first group concerns the *query system*, that is structured in the molecular components, the molecular systems and the cellular systems level. The second section concerns the *data analysis tools *available through the web interface. This area includes: the "Neighbourhood density and hubs" and the "Shortest paths" tools, both relying on the PPIs network analysis; the gene-annotation enrichment analysis (GEA); the Blast tool [28], which aligns query sequences against the data stored in the G2SBC Database. Finally, the web interface maintains a *model oriented section*, which involves two aspects. The first one concerns the interaction among cell cycle regulation and breast cancer: due to this connection it is possible to retrieve the breast cancer genes involved in cell cycle control and simulate the associated mathematical models. The second regards the mathematical models related to carcinogenesis, tumour growth and response to treatments.

### The query system

At the molecular components level, it is possible to query the G2SBC Database through gene or protein identifiers, synonyms and descriptions, in order to directly retrieve the breast cancer gene report. In this page users can find the information related to each gene that has been integrated into the database. This data includes gene and gene product identifiers, SNPs, molecular alterations involved in breast cancer, microRNAs, tissue expression, drugs, molecular interactions established by gene products, molecular pathways regulated by the listed protein and cell cycle associated kinetic models.

Moreover, it is possible to query the system starting from information about gene products function, represented by the protein domains and the GO molecular functions. An interesting application of this query concerns the knowledge about proteins involved in a key biological function, which is often under aberrant control in cancer conditions: transcription regulation. In fact, through this query, it is possible to list the transcription factors associated with molecular alterations in breast cancer.

Another possibility is to query the system considering the spatial localisation. This information includes the knowledge related to the gene localisation within the genome by means of spatial coordinates: in this case, the G2SBC Database provides the interactive visualisation of the genome by means of the UCSC Genome browser [29]. Spatial localisation concerns also the cell environment, a feature accomplished by using the GO cellular components annotation. An example is focusing on the membrane proteins, which play an important role at the beginning of intracellular signalling cascades and contribute, for instance, to the cancer cells insensitivity to anti-growth signals.

#### Molecular alterations

The G2SBC Database contains several types of molecular alterations associated with breast cancer. These alterations encompass the genome (mutations and SNPs), the transcriptome (RNA expression level and splicing variations) and the proteome (protein expression level, sequence, structure, localisation), as shown in Table 1. The whole number of genes affected by at least one molecular alteration is 2238, approximately the 9%-11% of the human genes. Due to the wide use of gene expression microarray technology, almost all of these genes show at least one alteration regarding the transcriptome. A lower number of genes present DNA and proteome variations.

The list of alterations associated with a particular gene is available in the gene report. Each alteration is annotated through some features - that vary according to the type of molecular alteration - such as the contig number, the direction of gene expression variation (up/down), the experimental method used, the cancer type or the cell line. Lastly, each molecular alteration is reported along with the reference to the paper where its experimental identification has been described.

#### Biochemical pathways and breast cancer genes

Considering the molecular systems layer, it is possible to query the G2SBC Database starting from a specific biochemical pathway name, exploiting data from KEGG, Reactome and GO BP. This system level query follows a *top-down *approach, which leads to the "building blocks" starting from the systems. In this context, the user interested in a particular biological process can retrieve the list of genes that may affect its normal activity leading to cancer conditions. An example is the response to lowered oxygen tension, an important process for tumour progression; searching through the keyword "hypoxia" it is possible to understand that the GO BP term "response to hypoxia" includes 30 genes which have at least one evidence of association with breast cancer.

#### Breast cancer types

For what concerns the information that can be retrieved from cellular and tissue levels, the query system lists a series of breast cancer types (e.g. ductal, lobular, medullar) and subtypes (e.g. ErbB2 positive, estrogen receptor positive). A specific breast cancer type can be selected in order to obtain the list of genes products (i) found expressed in the considered breast cancer type according to the HPA data and/or (ii) associated with molecular alterations identified in the selected breast cancer type.

**Figure 2:**
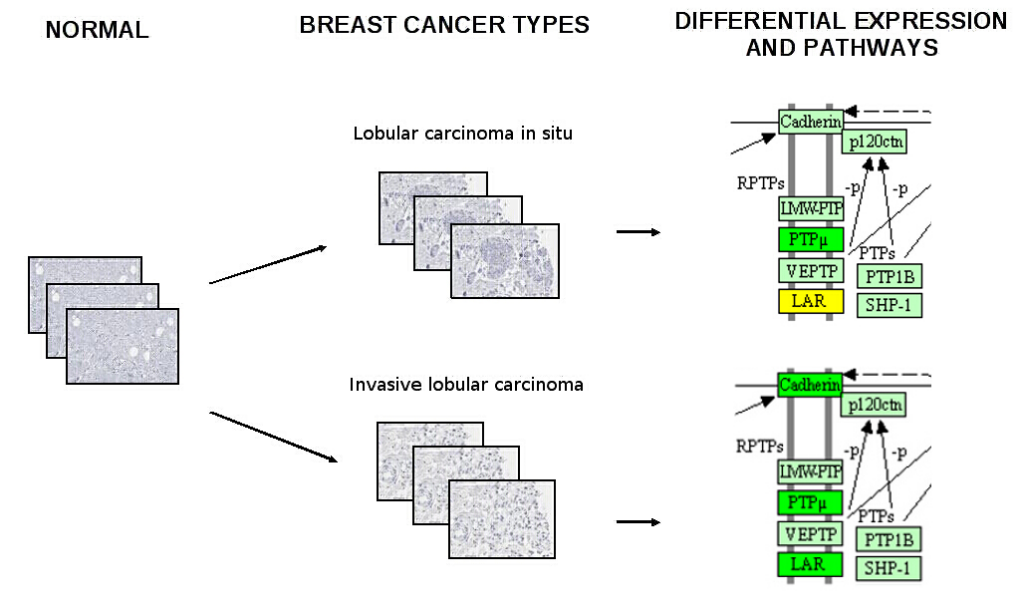
**Integration between protein expression in different breast cancer types and biochemical pathways data**. A use case showing the differential expression of some proteins belonging to the "adherens junction" KEGG map in lobular carcinoma in situ and invasive lobular carcinoma tissues images collected from the HPA database. Green: down-regulation; yellow: similar expression.

Breast cancer types supported by HPA data are provided with information concerning protein differential expression with respect to the normal tissue, Figure 2. For each protein, the number of tissue images where it has been up-/down-regulated or where it does not change is reported. This information has been integrated with KEGG Pathways: for each pathway, the number of the proteins, (i) that are up-regulated in the selected breast cancer, (ii) that show a similar expression level with respect to the normal tissue, (iii) that are down-regulated in the selected breast cancer, is reported. These data can be easily visualised by following the link provided to KEGG maps images, where proteins are automatically highlighted using distinct colours, according to their expression (i.e. red indicates up-regulation while green indicates down-regulation).

In this context, the multilevel data integration realised by the G2SBC Database provides some insights on the characterisation of the different breast cancer types in terms of genes and biochemical pathways.

#### Ontologies improve the search of database content

Other than representing an important instrument for generating new hypotheses and producing novel knowledge [30], the ontology layer which underlies the database structure improves the user capability of browsing data, due to the available standardised terms and the connection map provided by the ontology tree. GO terms and KEGG pathways are browsable through a section where the GO directed acyclic graph and the KEGG pathways hierarchy tree are available. Moreover, breast cancer genes have been mapped to the GO terms and the KEGG pathways: hence, it is possible to examine the number of breast cancer genes associated with each molecular function, cellular component, biological process and KEGG pathway. By querying all the genes associated with a specific GO term, it is possible to retrieve even the genes associated with terms that present an "is a" relationship with the initial one. For example, selecting the "damaged DNA binding" molecular function, both the terms "oxidised DNA binding" and "alkylated DNA binding", which describe particular types of damage, are retrieved thanks to the ontology layer.

### G2SBC Database embedded tools

The G2SBC Database provides a series of tools which can be used to analyse the database content. By means of the sequential use of these tools it is possible to create analysis pipelines, which in some cases can lead to new predictions.

#### Neighbourhood density, hubs and shortest paths

G2SBC Database web interface provides two tools that rely on the application of the graph theory to biological networks.

The first tool retrieves breast cancer genes that have a particular neighbourhood density in the PPIs network and establish a specific number of interactions. On one hand, a protein with a high neighbourhood density, measurable by means of the so called clustering coefficient, is potentially part of a protein complex [31] and, more generally, this feature may indicate that it belongs to a group of proteins that cooperate for a specific cell function. For instance, this is the case of the highly connected protein Polr2f (RNA polymerase II, polypeptide F), which establishes physical interactions with four polypeptides belonging to the RNA polymerase II and five subunits of the mediator complex, which is a co-activator involved in the transcription of nearly all RNA polymerase II-dependent genes [32]. On the other hand, proteins presenting a high number of interactions act as hubs in the PPIs network. These proteins represent elements which play an important role for the integrity of the network and are often associated with potential weak spots for cell functions [33]. The G2SBC Database allows the user to focus on hub proteins for which literature evidences of association with breast cancer are available.

The second tool concerns the "Shortest paths analysis", which is particularly useful in systems biology since it enables the reconstruction of biochemical pathways (an example of the use of shortest paths for the reconstruction of metabolic networks can be found in [34]). The G2SBC Database provides a tool for calculating the shortest path in the PPIs network between two breast cancer proteins. The result of this calculation is a table that lists the proteins belonging to the path and the biochemical pathways in which they are involved. This integration enables the user to clarify the relationships between breast cancer genes on the basis of the molecular systems in which they are included. For instance, this tool shows that proteins encoded by the genes CCND1 (cyclin D1) and EGFR (epidermal growth factor receptor), both associated with breast cancer, have a distances on the PPIs equal to 2, due to their common interacting partner STAT3 (signal transducer and activator of transcription 3). Moreover, it is possible to observe that all the three proteins have been annotated as members of the "Pathways in Cancer" map of KEGG Pathway.

#### Gene-annotation enrichment analysis (GEA) enables the query result characterisation

**Figure 3:**
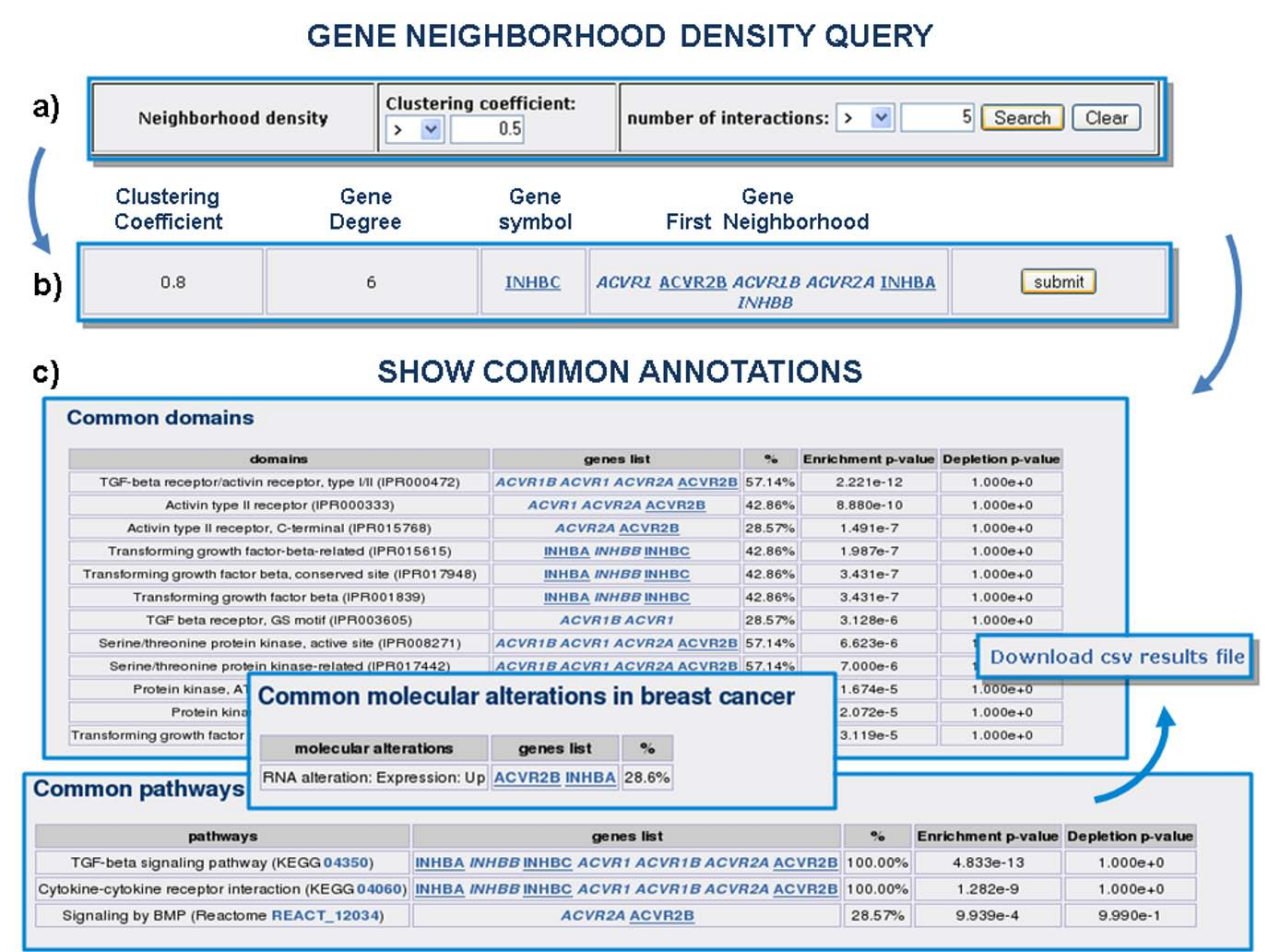
**An example of analysis in which the G2SBC Database tools are combined**. a) Query based on the clustering coefficient of breast cancer proteins in the PPIs network. I) One row of the results: the breast cancer gene INHBC has a high clustering coefficient (0.8) and establishes 6 interactions (degree). c) INHBC along with the genes in its first neighbourhood in the PPIs network are submitted to the GEA tool (only part of the results are shown).

The G2SBC Database provides a tool for GEA. The application finds the set of annotations associated with the input gene set. Each annotation term is coupled with a *P *value (calculated through the appropriate cumulative hypergeometric distribution) indicating the probability of obtaining the observed distribution of terms among the whole gene set in a hypergeometric experiment. This measure identifies annotation terms that are significantly enriched in a specific gene set. These terms represent distinctive properties of the gene set and constitute the starting point for further functional characterisation. The GEA tool is integrated in the web interface of G2SBC Database and is available whenever a gene list is obtained. Results can be downloaded in a text file. For instance, by performing an appropriate query on the clustering coefficients and the number of interactions, the user discovers that the protein inhibin, encoded by the gene INHBC, is part of a highly interconnected module of proteins which involves the activins (ACV proteins), as shown in Figure 3. Both inhibins and activins are growth factors involved in cell differentiation and proliferation [35]. By running the GEA tool on the set of proteins involved in the module the user can find, for example, that terms like "TGF-beta signalling pathway" and "cytokine-cytokine receptor interaction" have significant *P *values and, hence, these terms represent the annotation signature that characterises the gene set.

It is worth noting that the GEA tool is useful whenever the selected breast cancer gene is poorly annotated. In this case, the tool provides a way to study the annotations of its interacting partners and gain some insights, according to the concept of network-based function prediction [36] (the closer the proteins in the PPIs network, the higher the functional similarity). For example, the gene DCN (decorin) is currently associated with two GO BP terms: a very general term, "organ morphogensis", and "peptide cross-linking via chondroitin 4-sulfate glycosaminoglycan". The GEA tool indicates that DCN protein interacting proteins regulate "collagen fibril organisation" (*P *value 3.66E-8), "skin morphogenesis" (*P *value 4.46E-06), "skeletal system development" (*P *value 8.02E-6), a series of annotations which suggest more detailed biological roles of DCN. Another case is represented by the gene MTA1 (metastasis associated 1), that is currently annotated with two general GO BP terms: "regulation of transcription, DNA-dependent" and "signal transduction". The analysis of MTA1 protein neighbours shows that it interacts with proteins that regulate "histone deacetylation" (*P *value 2.253E-5), "nucleotide-excision repair, DNA damage removal" (*P *value 6.05E-5) and "chromatin modification" (*P *value 8.89E-5), that are all processes related to the DNA transcription.

#### SNP mutation structural modelling

For each SNP annotated in a breast cancer gene exon, which leads to missense in the corresponding protein, a pipeline aimed at modelling the macromolecule with the allele variant is available. In detail, the gene report provides an application that shows the gene structure where the annotated SNPs in the CDS (coding DNA sequence) are highlighted. By selecting the desired SNP, the web interface lists the IDs of the available PDB structures whose chains contain a model of the wild type sequence associated with the selected allele variant. The user can choose one chain and the complete structure is automatically downloaded from the PDB web site. Starting from this PDB structure, the polymorphic model is created using an automatic approach based on a customised python script from Modeller [37]. It replaces the side chain of the polymorphic residue in the PDB file and optimises the conformation by energy minimisation and molecular dynamics. After the computation, the PDB formatted structure with the polymorphic residues highlighted is provided through the web interface.

#### Genes distribution among breast cancer types

This tool provides a statistical approach to tissue-related information. Tissue data maintained in the database, which allows vertical integration among proteomic and cellular levels, are analysed by considering the association between genes and breast cancer types. By means of the data collected in the HPA, we calculated the frequency of detection of a specific gene product among the breast cancers types, in order to show its distribution over them. This content is shown in a dedicated web page reachable from the gene report, where the list of the antibodies exploited to detect the specific gene product is provided, together with a pie-chart plot which visualises the calculated percentage distribution.

### Mathematical models

**Figure 4:**
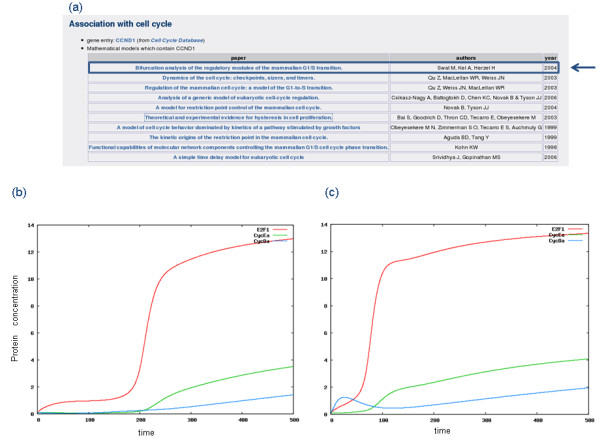
**Differential dynamics of a cell cycle ODE model **[41]** in normal and cancer states**. a) List of the cell cycle models that involve the cyclin D, which is often over-expressed in breast cancer cells; the model chosen for the simulation is indicated by the blue arrow. I) Normal cell cycle dynamics are characterised by a timed activation of the transcription factor E2F and the following activation of cyclin E in its active form (CycEa). c) Breast cancer state: according to the breast cancer associated up-regulation of cyclin D, its synthesis rate is increased, determining an earlier entrance into the S phase, with the early activation of the transcription factor E2F from which the cyclin E synthesis depends; such events determine a faster cell cycle progression allowing a more efficient tumour proliferation.

Since the cell cycle process is often affected by misregulations that can lead to cancer onset [1], the G2SBC Database has been coupled with the CellCycle Database. In particular, using the G2SBC Database it is possible to obtain the list of breast cancer genes that play a role in the cell cycle process and which take part in the mathematical models that describe the dynamics of this process in terms of temporal evolution of protein concentrations. This integration enables the analysis of cell cycle dynamics in breast cancer conditions according to the reported molecular alterations. The simulation of cell cycle models is run from the CellCycle Database web site. For instance, according to some evidences reported in the literature [38-40] and available in the G2SBC Database, the cell cycle gene corresponding to cyclin D has been found over-expressed in breast cancer cells. An example of this analysis, based on the cell cycle model described in [41], is presented in Figure 4. The entrance in the cell cycle S (synthesis) phase is represented through the dynamics of both the transcription factor E2F and cyclin E. In order to simulate the pathological state described in the G2SBC Database (cyclin D overexpression), it is possible to select the model (Figure 4a) and initialise it with a higher value of the cyclin D initial concentration. The computed dynamics reveals an earlier and faster entrance in the S phase, represented by a higher concentration of both the transcription factor E2F and cyclin E with respect to the normal state (Figure 4b,4c). 

Moreover, some mathematical models related to breast cancer development and response to treatment have been encoded in the Systems Biology Markup Language (SBML) [42] in order to allow their simulation in the dedicated section of the G2SBC Database. For instance, in the model by Spencer et al. [43] the multistep process which describes the transformation of a normal cell into a cancer cell through the acquisition of mutations is encoded in a set of ordinary differential equations; so doing, the model explores the role of angiogenesis, cell death rates, genetic instability, and replication during the cancer development. In the model section of the G2SBC Database it is possible to get general information about the mathematical models, and to access the abstracts of the articles where the models have been presented to scientific community. In this section, users can also explore the mathematical formalisation, simulate the model dynamics with respect to time, plot and download the simulations results.

## Conclusions

The G2SBC Database is a freely available resource developed with the aim of supporting research on breast cancer in a systems biology context. Using data integration a large amount of records have been collected in this database: therefore, enhanced query solutions and web tools are provided to infer non trivial knowledge, e.g. the proteins differentially expressed in each breast cancer type. The contents and tools provided encompass molecular components, molecular systems and cell systems layers. The G2SBC Database provides systems level queries (enabling a top down approach), tools based on PPIs network (e.g. the shortest paths) and a mathematical models section. Due to these features, it is possible to overcome the limits of a resource dedicated only to data exploration, enabling predictions that may induce new experiments.

The G2SBC Database will be periodically updated according to the publication of new research, biological data and mathematical models about breast cancer. We plan to integrate data from other sources, e.g. the Human Protein Reference Database (HPRD) [44] , and to enrich the mathematical model section. Moreover, we want to extend the system in order include data about *M. musculus *and *R. norvegicus*, making cross-species comparisons possible.

## Availability and requirements

The G2SBC Database is freely available at the URL http://www.itb.cnr.it/breastcancer.

## Authors' contributions

EM implemented the programs for data integration, PPIs network analysis, developed the web interface and the database; RA implemented the database, the web interface; IM implemented the SNP-related contents, developed the sequence and structure retrieving system and provided a complete overview on the work; FV created the ontological layer, the tissue contents and the molecular alterations section; AC contributed to data integration development; LM managed and directed the work. All authors read and approved the final manuscript.
